# Integrin α6β4 Upregulates Amphiregulin and Epiregulin through Base Excision Repair-Mediated DNA Demethylation and Promotes Genome-wide DNA Hypomethylation

**DOI:** 10.1038/s41598-017-06351-4

**Published:** 2017-07-21

**Authors:** Brittany L. Carpenter, Jinpeng Liu, Lei Qi, Chi Wang, Kathleen L. O’Connor

**Affiliations:** 1 Markey Cancer Center, University of Kentucky, Lexington, 40506-0509 USA; 2 Department of Molecular and Cellular Biochemistry, University of Kentucky, Lexington, 40506-0509 USA; 3 Department of Biostatistics, Division of Cancer Biostatistics, University of Kentucky, Lexington, 40506-0509 USA

## Abstract

Aberrant DNA methylation patterns are a common theme across all cancer types. Specific DNA demethylation of regulatory sequences can result in upregulation of genes that are critical for tumor development and progression. Integrin α6β4 is highly expressed in pancreatic carcinoma and contributes to cancer progression, in part, through the specific DNA demethylation and upregulation of epidermal growth factor receptor (EGFR) ligands amphiregulin (AREG) and epiregulin (EREG). Whole genome bisulfite sequencing (WGBS) revealed that integrin α6β4 signaling promotes an overall hypomethylated state and site specific DNA demethylation of enhancer elements within the proximal promoters of AREG and EREG. Additionally, we find that the base excision repair (BER) pathway is required to maintain expression of AREG and EREG, as blocking DNA repair molecules, TET1 GADD45A, TDG, or PARP-1 decreased gene expression. Likewise, we provide the novel finding that integrin α6β4 confers an enhanced ability on cells to repair DNA lesions and survive insult. Therefore, while many known signaling functions mediated by integrin α6β4 that promote invasive properties have been established, this study demonstrates that integrin α6β4 can dramatically impact the epigenome of cancer cells, direct global DNA methylation levels toward a hypomethylated state, and impact DNA repair and subsequent cell survival.

## Introduction

Integrins contribute to essential components of tumor progression such as survival, proliferation, and cell motility^1^. Specifically, integrin α6β4 is a known driver of tumor cell invasion^2^, which in turn promotes metastasis^3^. In cancer cells, integrin α6β4 signaling is activated upon binding to laminin extracellular matrix proteins and in cooperation with growth factor receptors such as EGFR, RON, and c-MET^4–6^. Activation of integrin α6β4 results in stimulation of downstream signaling pathways including PI3K, MAPK, Src family kinases, Rho family small GTPases, and the Nuclear Factor of Activated T-cells (NFAT)^7–9^ that contribute to invasion, angiogenesis, anoikis-resistance, cell survival, and proliferation^10^. Integrin α6β4 enhances these properties in part through transcriptional upregulation of pro-tumorigenic genes including S100A4 in breast cancer^11, 12^ and the EGFR ligands AREG and EREG in pancreatic carcinomas^13^.

The importance of AREG and EREG in tumor progression, therapeutic resistance, and as a potential prognostic and predictive biomarker has been well established in multiple cancer types^14, 15^. Cleavage of pro-AREG and pro-EREG by the MMPs results in protein release and autocrine signaling to activate EGFR^13^. AREG and EREG are unique in their ability to cause EGFR recycling back to the plasma membrane for reactivation^16, 17^. EGFR signaling by AREG and EREG is enhanced in pancreatic carcinomas and contributes to the aggressive nature of the disease^18, 19^. We have shown that AREG and EREG are required for HGF-mediated migration and invasion in response to signaling from integrin α6β4, further demonstrating their importance to invasive properties of cancer cells^13^. We and others find that AREG^13^ and EREG^13, 20^ gene expression is controlled by DNA methylation. However, the mechanisms guiding the demethylation of these promoters have not been elucidated.

Transcriptionally silenced genes have a repressive epigenetic state that compacts chromatin. Repressive epigenetic marks include non-acetylated histones, lysine methylation at H3K27 and H3K4 and cytosine methylation at CpGs^21^. Active DNA demethylation is tightly regulated and requires a series of enzymatic reactions that proceed through the BER pathway. This mechanism of epigenetic alteration is likely responsible for upregulation of pro-tumorigenic genes, as it has been identified for dynamic, context dependent modification of DNA^22, 23^.

The ten-eleven translocation methylcytosine dioxygenase (TET1) is the first crucial step in DNA demethylation as this protein recognizes specific 5-mCs to be targeted for removal by DNA repair and conversion from 5-mC to 5-hydroxymethyl cytosine (5-hmC)^23^. 5-hmC can be further oxidized by TET proteins to 5-carboxycytosine (5-caC) and 5-formylcytosine (5-fmC); however, these derivatives are found less often in the genome, and their complete function is still being characterized^24^.

5-mC products are identified by growth arrest and DNA damage inducible alpha (GADD45A). GADD45A is responsible for recruitment of other repair factors to CpG sites for removal of methyl groups, and has been implicated as a necessary step in DNA demethylation by providing a link between epigenetics and DNA repair^25, 26^. GADD45A recruits Activation Induced Cytidine Deaminase (AID) and Apolipoprotein B mRNA Editing Enzyme, Catalytic polypeptide-like (APOBEC) proteins^26^, which deaminates 5-hmC to 5-hmU, generating a G-U DNA mismatch. This mismatch is removed by thymine DNA glycosylase (TDG) or methyl-binding protein 4 (MBD4). This cleavage activates the normal functions of the BER pathway including cleavage of the DNA backbone by AP-endonuclease and repair back to a non-methylated cytosine by XRCC-1, PARP-1, DNA ligase, and DNA polymerase^27^.

Here, we sought to determine in mechanistic detail how integrin α6β4 stimulates DNA de methylation of AREG and EREG by systematically examining the NER and BER pathways and define the impact of integrin α6β4 on genome-wide methylation patterns.

## Results

### Integrin α6β4 promotes laminin deposition in pancreatic cancer cells

**Figure 1:**
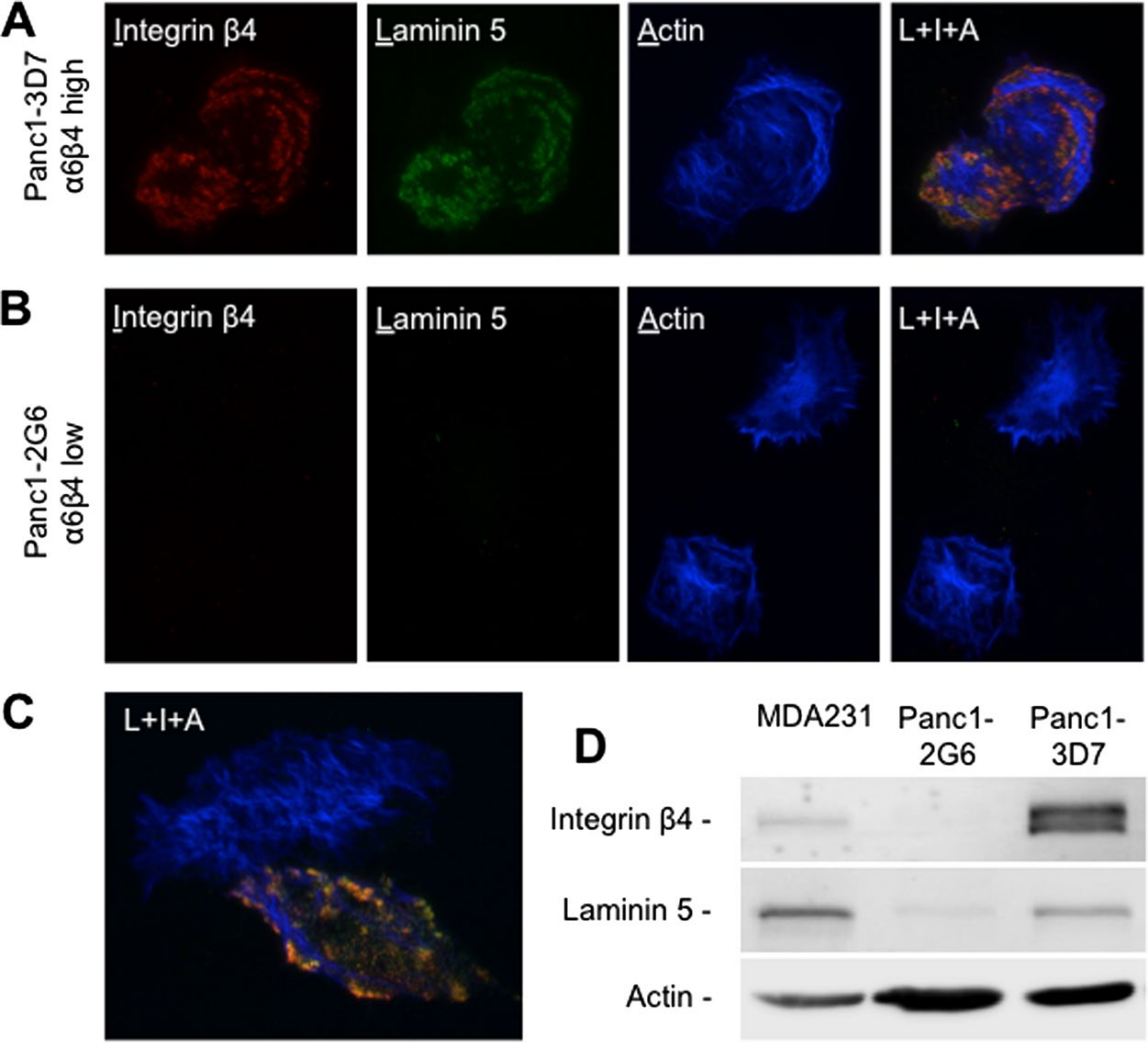
Integrin α6β4 binds to and promotes secretion of laminin-5 in Panc1 cells. (**A**–**C**) Panc1 clones 3D7 (A; high α6β4) and 2G6 (B; low α6β4) were plated on collagen I coated coverslips and allowed to adhere under normal culture conditions for 4 hours. Cells were fixed and immunostained for integrin β4 subunit (red), laminin-5 (γ2 subunit; green), or f-actin (blue) as described in the Methods section. Cells were imaged by TIRF microscopy using the same exposure times and settings. Pearson’s coefficient for colocalization between integrin β4 and laminin-5 were 0.8 for Panc1-3D7 (**A**) and 0.011 for Panc1-2G6 (**B**). These values are representative for the 30 cells analyzed for each cell line. (**C**) Represents a rare β4 expressing cell in the Panc-2G6 cell population. (**D**) Western blot analysis of whole cell extracts from MDA-MB-231 (positive control), Panc1-2G6 and Panc1-3D7 cells for integrin β4, laminin-5 and actin (loading control).

Integrin α6β4 signaling has been shown to be ligand-independent in several model systems where the integrin does not require exogenous ligand to mediate its effects^7^. In our previous studies, we have found that exogenous laminin is not required to see changes in gene expression associated with enhanced invasion and migration^13^. Interestingly, the Jones group has demonstrated that integrin α6β4 promotes secretion and deposition of laminin-5, a major laminin isoform implicated in integrin α6β4 signaling, in the extracellular matrix, which in turn promotes motility of keratinocytes^28^. To test if endogenous secretion of laminin-5 is responsible for ligating integrin α6β4 in the Panc1 model, we plated Panc1-2G6 (low α6β4) and Panc1-3D7 (high α6β4) cells were plated onto collagen-coated coverslips for 4 hours and stained for the laminin γ2 subunit, which is unique to laminin-5. We find that in integrin α6β4 high expressing Panc1-3D7 cells, there is enhanced deposition of laminin, which strongly colocalizes with the integrin β4 subunit (Fig. 1A), which is in line with observations from keratinocyte studies. In contrast, cells with low integrin α6β4 have low detectable levels of laminin (Fig. 1D), which is not deposited extracellularly (Fig. 1B), implicating a deficient integrin α6β4 signaling network. Likewise, our previous studies demonstrate the enhanced migratory abilities of integrin α6β4 high versus low expressing pancreatic cancer cell lines when plated on laminin^29^. We chose to use these stable subpopulations derived from the Panc1 cell line as our model system for studying the impact of integrin α6β4 on the transcriptome as we have clearly demonstrated both variable levels of the integrin α6β4 and its cognate ligand, laminin-5.

### Epigenetic events regulate expression of AREG and EREG

**Figure 2:**
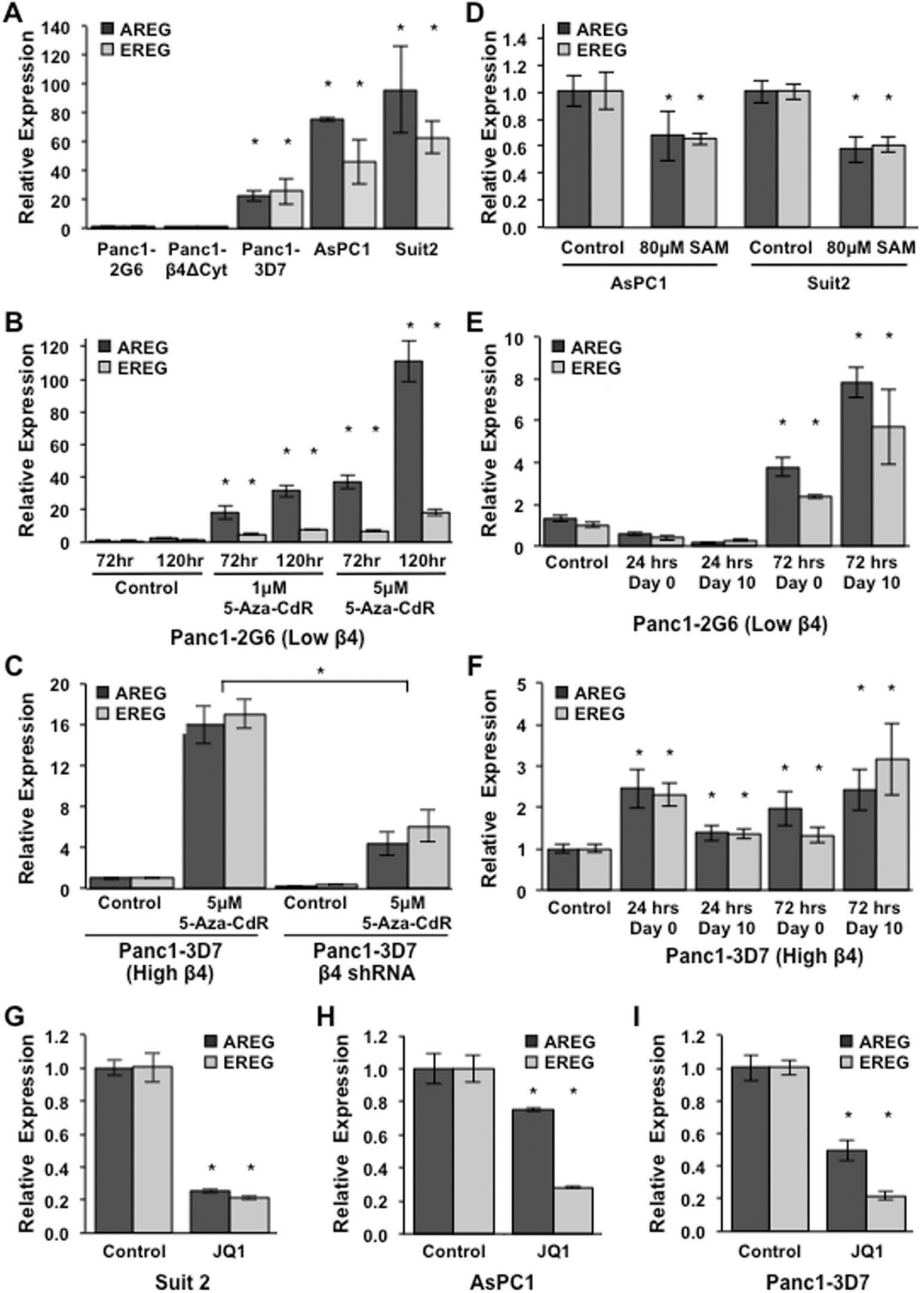
AREG and EREG expression is mediated by DNA demethylation in response to signaling from the integrin α6β4. (**A**) Expression of AREG and EREG was compared in Panc1-2G6 (low α6β4) and cells expressing a dominant negative α6β4 (Panc1-β4ΔCyt), Panc1-3D7, Suit2, and AsPC1 (high α6β4; in order of increasing expression) cell lines. (**B**) Panc1-2G6 cells (low α6β4) were treated with vehicle only (control) or with 1 μM or 5 μM 5-aza-2′-deoxycitine (5-aza-CdR) in fresh medium daily for 3 or 5 days. (**C**) Panc1-3D7 stably expressing an shRNA targeting the β4 subunit or a non-targeting (NT) shRNA control vector were treated with 2 μM 5-aza-CdR for 3 days and then assessed for AREG and EREG expression. (**D**) AsPC1 and Suit2 (high α6β4) were treated with vehicle only (control) or 80 μM S-adenosylmethione (SAM) in fresh medium daily for 5 days (**E**,**F**). Panc1-2G6 (**E**) and Panc1-3D7 (**F**) cells were treated with 2 μM 5-aza-CdR for 24 or 72 hours, 5-aza-CdR was removed and cells were either collected immediately or maintained in culture for 10 days. (**G**–**I**) Cells with high integrin α6β4 were treated with vehicle only (control) or 0.5 μM JQ1 overnight and harvested for analysis by QPCR. For all experiments RT-PCR was used to convert RNA to cDNA and QPCR was used to assess AREG and EREG expression. Data depicted here are representative of at least three different experiments and represent the mean +/− standard deviation. Statistical significance was calculated using a one-tailed t-test in which * denotes P < 0.05 as compared to controls, unless otherwise indicated.

Integrin α6β4 signaling stimulates progression of multiple types of cancer in part by altering the transcriptome. Notably, expression of AREG and EREG positively correlates with expression of and signaling through integrin α6β4 (Fig. 2A), supporting our previous work^13^. To determine if AREG and EREG expression is regulated by DNA methylation, Panc1-2G6 cells were treated with the DNA methyltransferase inhibitor 5-aza-CdR at indicated concentrations, harvested at indicated time points, and RNA analyzed by QPCR. We found that both AREG and EREG mRNA expression increased in a time and dose dependent manner (Fig. 2B) demonstrating the susceptibility of AREG and EREG to DNA methylation. Furthermore, integrin α6β4 was required for induction of AREG and EREG mediated by 5-aza-CdR, as knocking down the integrin β4 in Panc1-3D7 cells hindered epigenetic induction of AREG and EREG expression (Fig. 2C). Considering that epigenetic changes are reversible, AsPC1 and Suit2 cells, high expressers of integrin α6β4, AREG, and EREG, were treated with the methyl donor S-adenosylmethionine (SAM) and assessed for AREG and EREG expression by QPCR. These data revealed a 50% decrease in expression of AREG and EREG as seen in Fig. 2D. Taken together these data indicate that signaling from integrin α6β4 and DNA demethylation are required to drive AREG and EREG expression.

True epigenetic alterations are stable changes maintained across many generations. Since 5-Aza-CdR can modify the epigenetic landscape^30^, we assessed the impact of short term 5-aza-CdR treatment on AREG and EREG expression by treating cells with the indicated concentrations of 5-Aza-CdR for 24 or 72 hours. 5-Aza-CdR was removed and cells were either harvested immediately or maintained in culture for 10 days. As shown in Fig. 1E, expression of AREG and EREG was not only induced 20–40 fold and maintained in Panc1-2G6 cells following 5-aza-CdR treatment but continued to increase when kept in culture 10 days post 5-aza-CdR removal. Treatment of Panc1-3D7 cells only slightly increased transcription of AREG and EREG (Fig. 2F), suggesting these stable epigenomic modifications have already taken place. These data confirm that the integrin α6β4 contributes to the stable upregulation of pro-tumorigenic molecules AREG and EREG through epigenetic alterations.

Alterations in DNA methylation strongly impact the activity of enhancers, which activate specific transcriptional profiles through recruitment of transcription factors that interact with the mediator complex^31^. To determine if enhancer activity is required for AREG and EREG expression in pancreatic cancer cells, we treated cells with JQ1, a BET bromo-domain inhibitor that is specific for BRD4^32^. BRD4 interacts with the elongating factor P-TEFB in Pol II complexes to enhance transcription for both protein-coding and enhancer-derived noncoding RNAs^33^. We found that AREG and EREG expression markedly decreased with JQ1 treatment, thus indicating their transcriptional dependence on enhancer function (Fig. 2G–I).

### Integrin α6β4 impacts genome wide DNA methylation patterns

**Figure 3:**
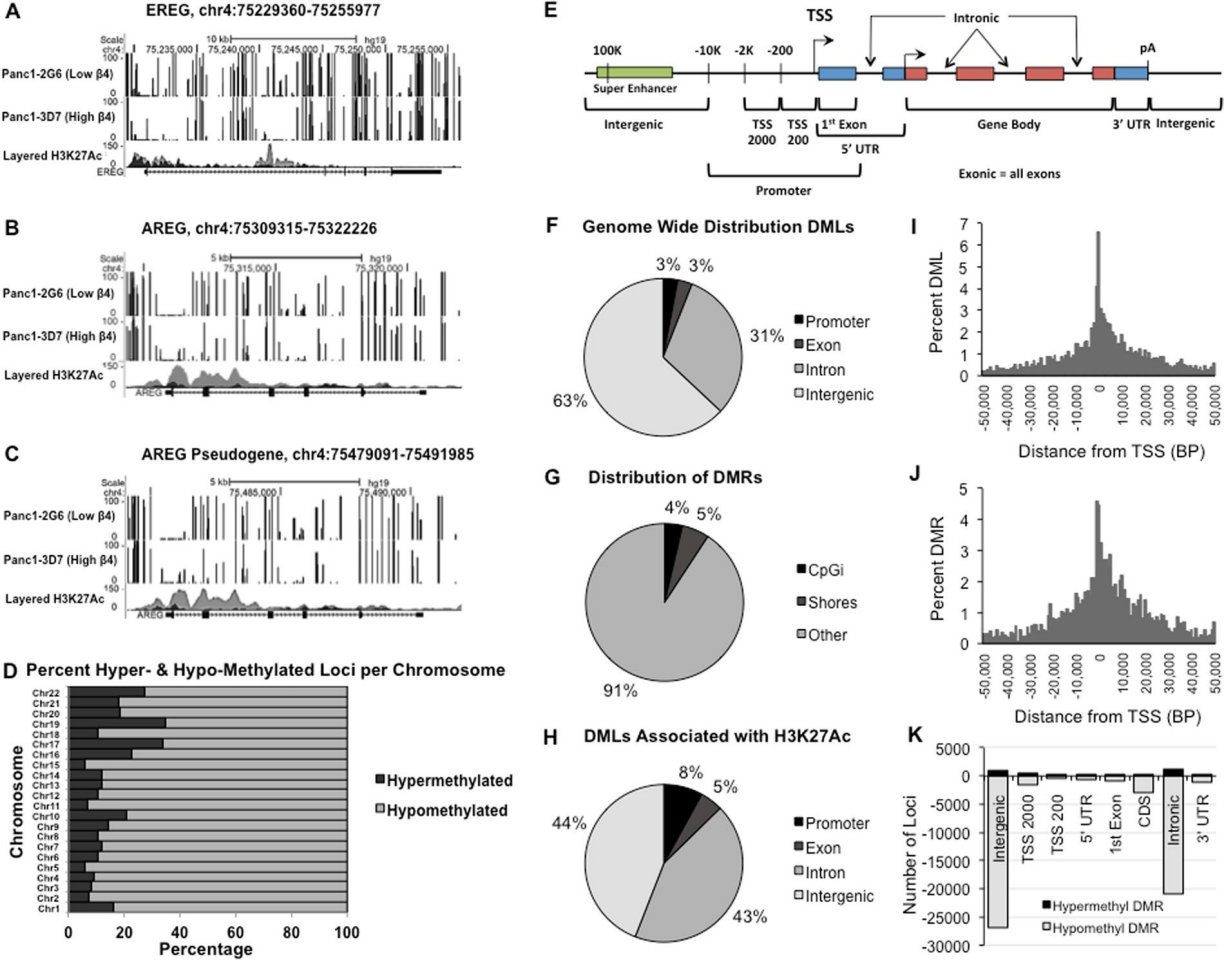
The integrin α6β4 drives both gene specific and global DNA hypomethylation. (**A**–**C**) Genomic DNA from Panc1-2G6 (β4 low; upper panels) and Panc1-3D7 (high β4; lower panels) was processed for high-resolution methyl-seq by the NextGen Sequencing Core at the Norris Comprehensive Cancer Center. Samples were analyzed bioinformatically and percent methylation shown for EREG (**A**), AREG (**B**), and AREG pseudogene (**C**). (**D**) Percent hypomethylation and hypermethylation per chromosome when comparing Panc1-3D7 vs. Panc1-2G6. (**E**) Defined regions of interest assessed for changes in DNA methylation (**F**) Location of DMLs across the genome. (**G**) Percent of methylation changes located in CpG islands and shores. (**H**) Location of DMLs associated with H3K27Ac. (**H**) Distance from TSSs for DMLs (**I**) Distances from TSSs for DMRs (**J**) Number of both hypomethylated and hypermethylated regions corresponding to genomic features.

To define DNA demethylation changes that drive expression of AREG and EREG, sodium bisulfite conversion and whole genome sequencing was performed on genomic DNA from pancreatic cancer cells with either high (Panc1-3D7) or low (Panc1-2G6) integrin α6β4 expression. Sequencing reads were aligned to the reference genome, GRCH37, mapped to the AREG and EREG genes, and visualized using the UCSC genome browser. We found that cells with high integrin α6β4 (Fig. 3A and B; bottom panels) have reduced DNA methylation within intronic regions of both EREG (Fig. 3A) and AREG (Fig. 3B), confirming that the integrin α6β4 drives site-specific DNA demethylation, and defining the critical CpG sites of AREG and EREG that become altered downstream of integrin α6β4.

Importantly, we also found alterations in DNA methylation in an AREG pseudogene, which lies directly downstream of AREG (Fig. 3C). When examining these two regions, both the sequence structure and regulatory similarity were noted as they are 99% homologous when blasted against the reference genome. Since Bismark only reports unique matches, the multi-mapping scenario of AREG and its pseudogene made it difficult to investigate the methylation alternations in these two regions. However, the analysis was possible by masking AREG pseudogene and mapping AREG, and vice-versa for AREG pseudogene. We attempted to investigate this further by using bisulfite conversion with methylation specific PCR to confirm altered CpGs within this region. However, the sequence similarity between these two regions and difficulty designing unique primers for bisulfite converted DNA proved that this analysis was technically unfeasible.

Regions that had the greatest difference in DNA methylation in both AREG and EREG as a result of integrin α6β4 signaling corresponded to areas enriched in H3K27Ac marks (Fig. 3A,B), as annotated by the ENCODE project, that are reported to mark active enhancer elements^34^. Additionally, a super-enhancer associated with AREG and EREG expression lies between AREG and the AREG pseudogene^35^. We found no significant differences in super-enhancer DNA methylation (data not shown), indicating that it is unlikely that DNA methylation of this element is the major driver for enhanced AREG and EREG gene expression. Taken together, these data, along with our observation that BRD4 is required for AREG and EREG expression, indicate that DNA demethylation of enhancer elements localized within the proximal promoters of AREG and EREG drive expression in response to integrin α6β4 signaling.

Next, we examined the genome wide effects of integrin α6β4 on DNA methylation using our WGBS data. A total of 236,371 differentially methylated loci (DML; 207,168 hypomethylated and 29,203 hypermethylated) were identified comparing Panc1-3D7 vs. Panc1-2G6. Figure 3D illustrates the percentage of hypermethylated and hypomethylated events per chromosome as a percent of the number of DMLs. Of the DMLs identified, 87.6% were hypomethylated and 12.4% were hypermethylated, thus indicating that the integrin α6β4 shifts chromatin to a more hypomethylated state. Further analysis of these data revealed that only 3.1% of these loci were located in promoter regions, 2.1% in exonic regions, 31.1% in intronic regions and 63.1% were in intergenic regions (Fig. 3F). 13,889 differentially methylated regions (DMRs) were identified, of which only about 4% were located in CpG islands, and 5% in CpG shores (Fig. 3G). We found that 40,609 DMLs associated with H3K27Ac marks were hypomethylated as opposed to 13,679 DMLs hypermethylated. These events correspond to 4993 genes that have alterations in methylation within enhancer elements. As seen in Fig. 3H, the majority of these altered DML are localized to intronic and intergenic regions (defined in Fig. 3E) of which the majority are hypomethylated (Fig. 3K). This observation is typical of enhancer elements, as many enhancers are part of non-coding regions of the genome^36^. Additionally, we found that DMLs and DMRs occur predominantly within the first ten thousand base pairs on either side of the TSS with slightly more occurring after the TSS, as expected (Fig. 2I,J).

### AREG and EREG expression is not regulated by NER

**Figure 4:**
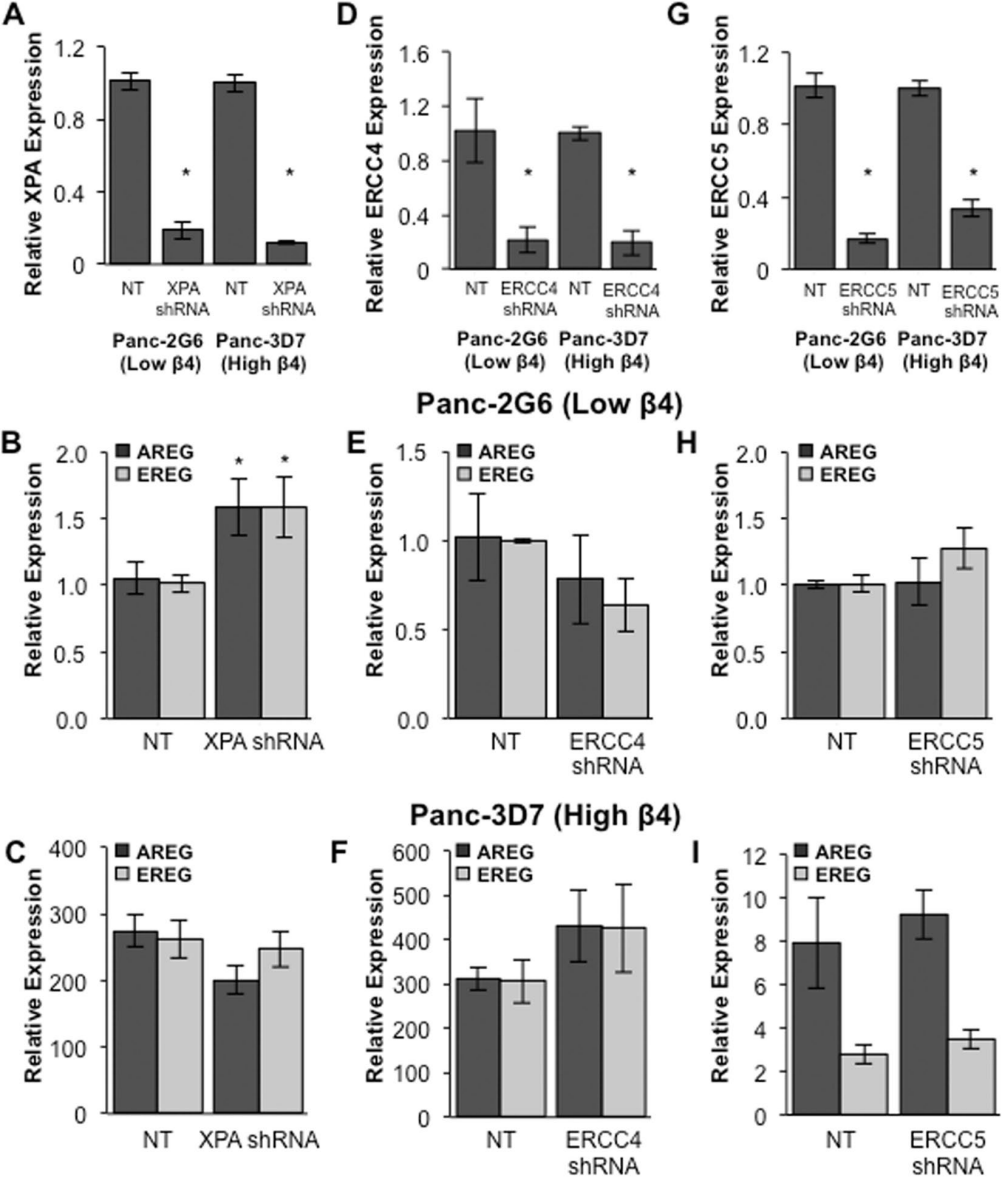
NER is not required for expression of AREG and EREG. Using lentiviral transfection stable knockdown of XPA (**A**), ERCC4 (XPF) (**D**), and ERCC5 (XPG) (**G**) was achieved in Panc1-2G6 (low α6β4) and Panc1-3D7 (high α6β4) cells as confirmed by QPCR. AREG and EREG expression was examined following knockdown in cells with both low α6β4 (**B**,**E**,**H**) and high α6β4 (**C**,**F**,**I**) expression. Data depicted are representative of at least three different experiments and represent the mean +/− standard deviation. Statistical significance was calculated using a one-tailed t-test in which * denotes P < 0.05 as compared to controls.

Since our data suggest that AREG and EREG DNA demethylation is an active process, we tested the hypothesis that DNA repair is required to maintain their expression downstream of integrin α6β4 signaling. The NER pathway, including the Xeroderma pigmentosum complementation group proteins XPA, XPG, and XPF, has been implicated in active DNA demethylation by DNA repair^37, 38^. Accordingly, we targeted molecules critical for and specific to the NER pathway and examined their impact on AREG and EREG expression. When knockdown of XPA (Fig. 4A) was achieved, transcription of AREG and EREG in Panc-3D7 (high α6β4; Fig. 4C) remained unaffected. AREG and EREG transcription in Panc-2G6 (low α6β4; Fig. 4B) showed a statistically significant increase when XPA was knocked down, which implies negative regulation. However, due to very low basal expression of AREG and EREG in these cells (cT value >35) it is unlikely to be biologically significant. Using specific shRNAs we knocked down ERCC4 (XPF; Fig. 4D) and ERCC5 (XPG; Fig. 4G) and demonstrated that effective knockdown of NER genes had relatively little or no effect on AREG and EREG expression (Fig. 4E–F,I–J). Taken together these data indicate that NER is not required to maintain AREG or EREG expression.

### Alterations in BER impact AREG and EREG expression

**Figure 5:**
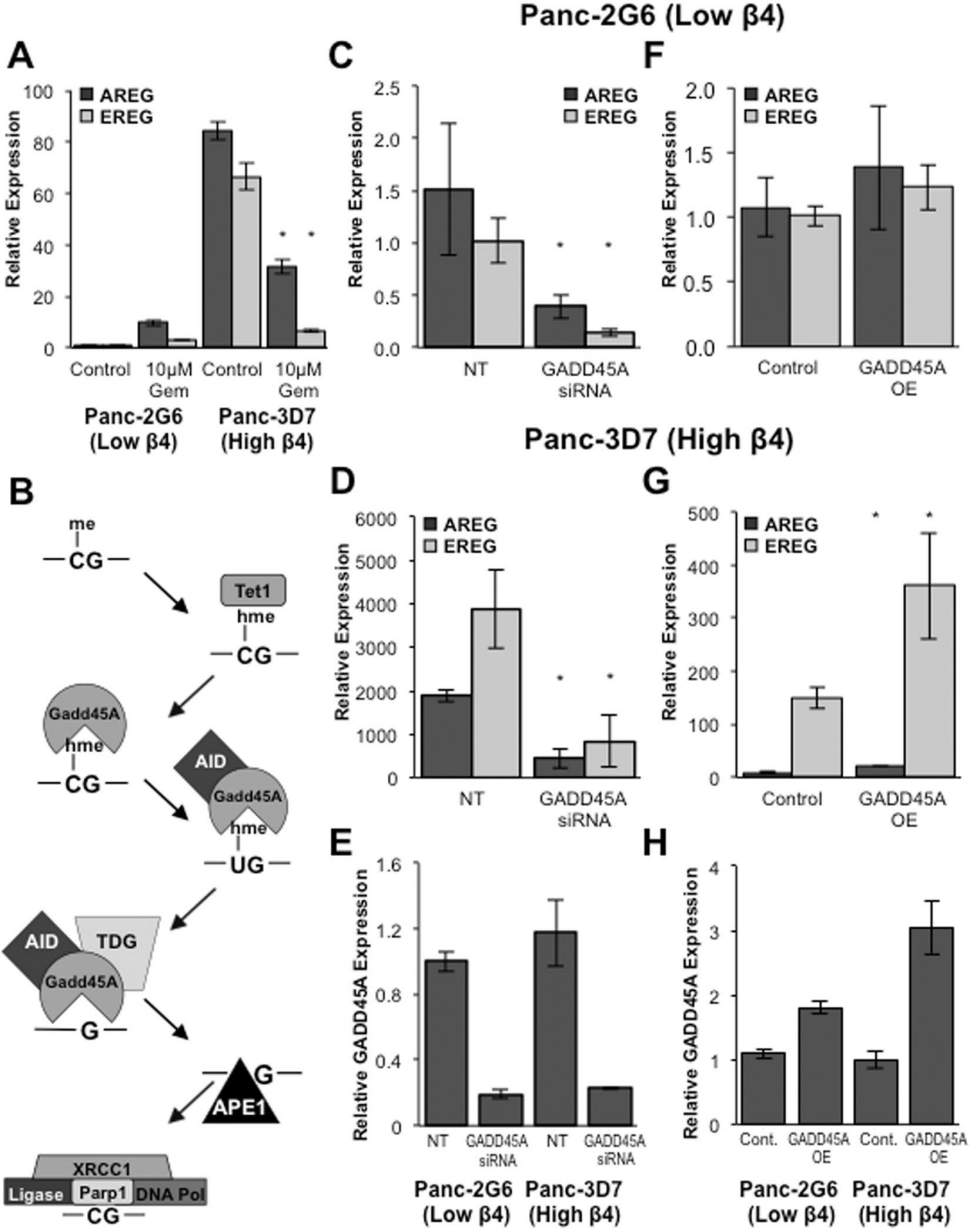
GADD45A is both required for and the rate-limiting step in activation of AREG and EREG expression. (**A**) Cells were treated with 10 μM Gemcitabine for 72 hours and expression of AREG and EREG measured by QPCR. (**B**) Summary of current literature for how GADD45A mediated DNA demethylation is achieved. Transient knockdown of GADD45A was achieved using electroporation and specific siRNA (**E**). Adenovirus was used to overexpress GADD45A in Panc1-2G6 and Panc1-3D7 cells (**H**). Changes in AREG and EREG expression were measured by QPCR in Panc1-2G6 (**C**,**F**) and Panc1-3D7 (**D**,**G**). Data depicted here are representative of at least three different experiments and represent the mean +/− standard deviation. Statistical significance was calculated using a one-tailed t-test in which * denotes P < 0.05 as compared to controls.

Gemcitabine is a chemotherapeutic with multiple proposed mechanisms of action, including depletion of deoxynucleotide triphosphates that are necessary for DNA synthesis and completion of DNA repair^39^. Interestingly, gemcitabine has been shown to specifically inhibit GADD45A mediated gene activation via DNA demethylation and DNA repair^40^. To investigate the role of DNA repair in expression of AREG and EREG, cells were treated with 10 μM gemcitabine for 72 hours. As demonstrated in Fig. 5A, AREG and EREG expression dramatically decreased in cells with high integrin α6β4 in response to treatment, thus indicating that DNA repair is required to maintain expression. As summarized in Fig. 5B, GADD45A mediated active DNA demethylation is achieved through BER. Therefore, we next investigated the role of TET1, GADD45A, TDG, and PARP1 in the regulation of AREG and EREG as key regulators of DNA repair-mediated DNA demethylation.

GADD45A is responsible for identifying residues for DNA demethylation by DNA repair^25, 41^. We modulated GADD45A in pancreatic cancer cells using either siRNA to knockdown or adenoviral infection to overexpress GADD45A and examined the effects on AREG and EREG expression. As depicted in Fig. 4, knockdown of GADD45A (Fig. 5E) resulted in decreased expression of AREG and EREG regardless of integrin α6β4 expression (Fig. 5C,D). Similarly, overexpression of GADD45A (Fig. 5H) resulted in a further increase in AREG and EREG expression, only in Panc1-3D7 cells (Fig. 5F vs G). These data indicate that GADD45A is a required for and is potentially a rate-limiting step in gene activation of AREG and EREG downstream of integrin α6β4 signaling.

**Figure 6:**
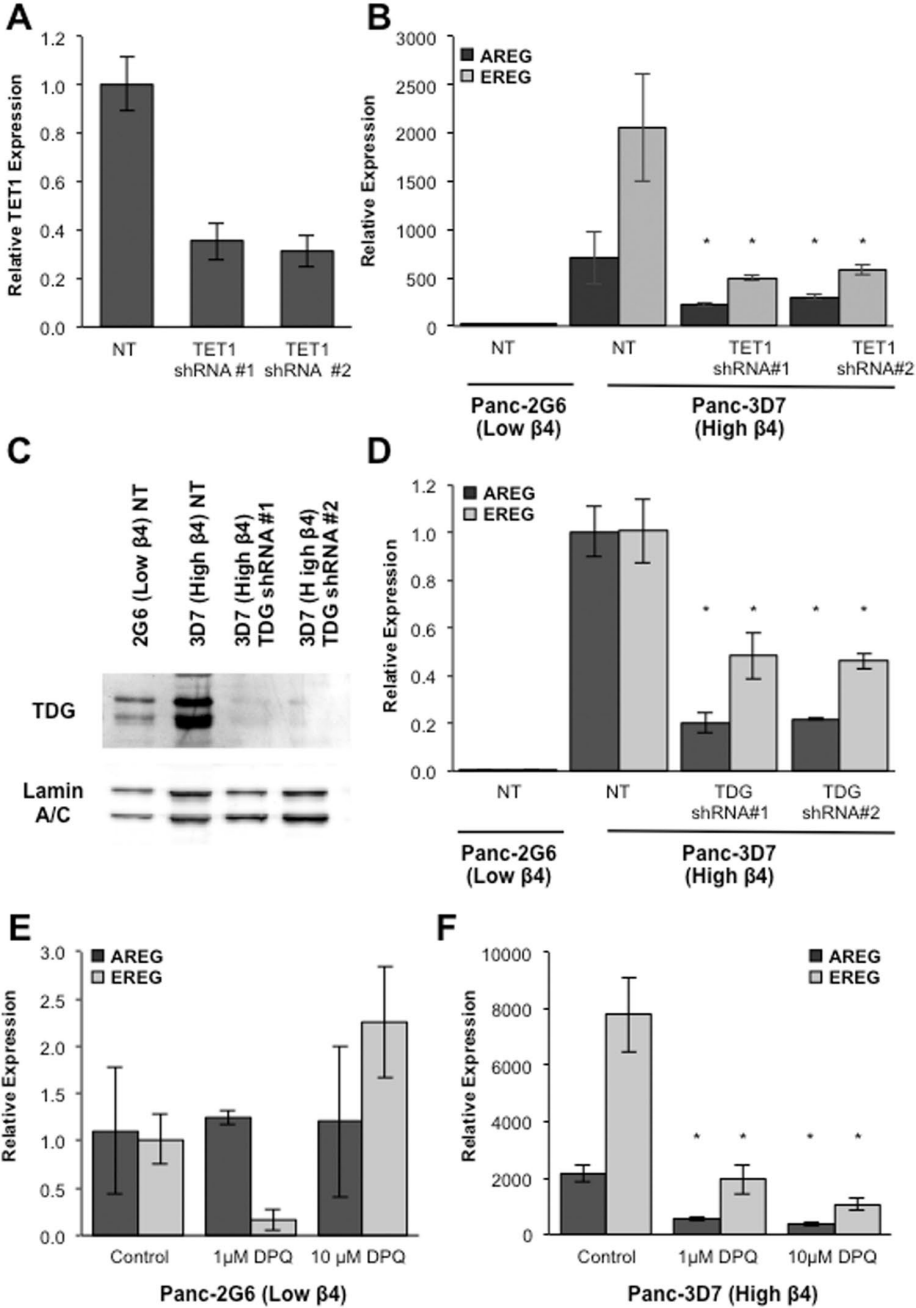
BER is necessary for induction of AREG and EREG expression downstream of integrin α6β4 signaling. (**A**,**B**) RNA was isolated from Panc1-3D7 cells stably expressing non-targeting or shRNA specific for TET1. QPCR analysis was used to confirm TET1 knockdown (**A**) and expression of AREG and EREG (**B**). (**C**) Nuclei were isolated from Panc1-2G6, Panc1-3D7, and Panc1-3D7 cells expressing specific lentiviral shRNA for TDG. Western blot analysis was performed on nuclear fractions for TDG and Lamin A/C used as a loading control. (**D**) Cells were collected and AREG and EREG expression measured by QPCR. (**E**,**F**) Cells were treated with either 1 μM or 10 μM 3,4-Dihydro-5-[4-(1-piperidinyl)butoxyl]-1(2 H)-isoquinolinone (DPQ) for 72 hours. Expression of AREG and EREG was measured by QPCR in Panc-2G6 (low α6β4; **E**) and Panc-3D7 (high α6β4; **F**) cell lines. Data depicted are representative of at least three different experiments and represent the mean +/− standard deviation. Statistical significance was calculated using a one-tailed t-test in which *denotes P < 0.05 as compared to controls.

TET proteins are solely responsible for oxidation of 5-mC to 5-hmC, 5-fC and 5-caC in mammalian DNA^42, 43^, which provide substrates for further processing to a cytosine by the DNA glycosylases and BER^44, 45^, with 5-hmC being the most common^43^. To test the role of the TET proteins, we depleted TET1 using specific shRNAs in Panc1-3D7 cells (Fig. 6A). As demonstrated in Fig. 6B, AREG and EREG expression is robustly decreased following a 70% reduction in TET1.

TDG has been found in complex with AID and GADD45A in the context of active DNA demethylation and evidence exists that glycosylase activity is necessary for this process^45^. As shown in Fig. 6C, there was substantially lower nuclear TDG protein expression in Panc1-2G6 compared to Panc1-3D7. As seen in Fig. 6D, this stable knockdown of TDG resulted in marked downregulation of AREG and EREG in Panc1-3D7 cells, indicating that TDG is necessary to maintain expression of AREG and EREG downstream of integrin α6β4, potentially through preferential localization of TDG into the nucleus.

PARP-1 is required for BER and is implicated in genome-wide and locus specific active DNA demethylation in part through epigenetic regulation of TET1^46^. Using a PARP-1 inhibitor, DPQ, we observed a dramatic decrease in AREG and EREG expression in Panc1-3D7 cells (Fig. 6F). However, in Panc1-2G6 cells, expression of AREG and EREG was relatively unaffected by PARP-1 inhibition (Fig. 6E), indicating that PARP-1 is mediator of AREG and EREG induction regulated by the integrin α6β4.

### Integrin α6β4 mediates cell survival and repair upon DNA damage

**Figure 7:**
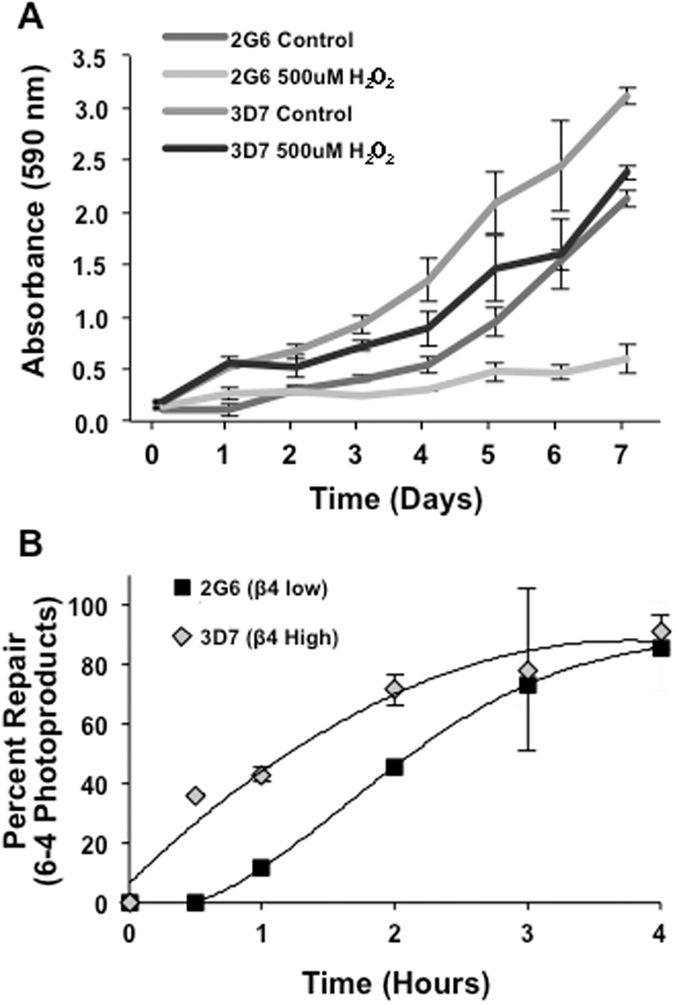
Integrin α6β4 promotes DNA repair and cell survival in response to DNA damage. (**A**) Cells were treated with 500 μM H_2_O_2_ in fresh medium daily for 7 days. Each day cell proliferation was measured by MTT colorimetric assay. (**B**) Cells were exposed to 30 J/m^2^ UV light and DNA isolated at indicated time points. Slot blot assay was performed using antibody for 6-4 photoproducts and percent repair compared to damage achieved immediately after exposure (0 hr).

We rationalized that if the integrin α6β4 is using the BER pathway to activate specific genes, the integrin may also enhance DNA repair in response to DNA damage. Therefore, we induced oxidative damage, which is repaired by the BER pathway, by exposing cells to 500 μM H_2_O_2_ over seven days and measuring cell viability by MTT assay. We observed a modest decrease in cell number in Panc1-3D7 cells; however, this H_2_O_2_ treatment nearly abolished Panc1-2G6 cells, indicating a decreased ability to survive insult by oxidative stress (Fig. 7A). To measure DNA repair more directly, we examined NER dependent DNA repair by exposing cells to 30 J/m^2^ UV light and measuring resolution of 6-4 photoproducts over time. As illustrated in Fig. 7B, Panc1-3D7 cells resolved UV induced lesions more rapidly than Panc1-2G6 cells, with a difference in half-life of about 1 hour. Taken together, these data indicate that the integrin α6β4 can both utilize DNA repair, and enhance the ability of cells to respond to, repair, and survive DNA damage.

## Discussion

While our knowledge of cancer epigenetics has developed rapidly, how dynamic epigenetic regulation is influenced by the tumor microenvironment to foster a metastasis phenotype has yet to be revealed. We find that integrin α6β4 is a critical mediator of DNA demethylation of two pro-invasive molecules, AREG and EREG. These specific changes in DNA demethylation of AREG and EREG occurred at enhancer elements within their proximal promoters that drive their expression downstream of integrin α6β4. Similarly, our data support integrin α6β4 as a modulator of genome-wide DNA methylation patterns, as overexpression of integrin α6β4 resulted in dramatic hypomethylation of the genome, with a significant percentage of these CpGs located in putative enhancer sites. Lastly, our study revealed that integrin α6β4 not only utilizes the BER DNA repair but also facilitates enhanced repair of DNA lesions, as cells with high integrin α6β4 survived better in response to oxidative stress, and directly repaired 6-4 photoproducts more rapidly. Our unique findings provide evidence that places integrin α6β4 as a critical mediator of cancer epigenetics, and thus offer new mechanisms for the integrin’s role in cancer progression.

Upregulation of invasion promoting molecules and subsequent activation of their downstream signaling targets are critical for the progression of cancer. Here, we demonstrate that AREG and EREG, which are established contributors of tumor progression^14, 15^, are upregulated downstream of signaling from integrin α6β4 and this upregulation is dependent on active DNA demethylation. This observation builds on our previous data showing that integrin α6β4 stimulates specific DNA demethylation of the S100A4 promoter, ultimately contributing to invasive capabilities of breast cancer cells^11^. Interestingly, work in squamous cell carcinoma and MDA-MB-231 breast cancer cells demonstrates that ECM content, cell-cell interactions, and 3D environment impact the methylation state of the E-cadherin promoter and this dynamic epigenetic plasticity helps drive EMT^47, 48^. These observations collectively solidify the role of the tumor microenvironment in regulating specific sites of DNA methylation, thus contributing to invasive growth of cancer cells.

Our analysis of genome-wide DNA methylation patterns revealed that integrin α6β4 dramatically reshapes the epigenetic landscape, shifting global DNA methylation patterns to a more hypomethylated state. Furthermore, this study shows that changes in specific CpG methylation within the AREG and EREG genes occurred in intronic regions that are not defined by the presence of CpG islands. These sites of altered DNA demethylation within AREG and EREG regulatory region correspond to known sites of H3K27Ac. Coupled with the requirement of BRD4 activity for AREG and EREG expression implicates the necessity for enhancer elements to drive gene expression. Our previous work on S100A4 yielded similar results as specific changes that control gene expression reside in an enhancer element located in a CpG rich region rather than a CpG island^11^. Similar to our gene specific data, most hypomethylation events induced by integrin α6β4 are not localized to CpG islands or promoter regions, but are instead found in intronic and intergenic elements. In addition, 23% of these regions corresponded to potential sites of H3K27ac, which is indicative of enhancer location^34^. These changes in DNA methylation are not surprising as hypomethylation of enhancer elements is tightly linked to overexpression of cancer promoting genes and gene profiles, as opposed to promoter methylation^49, 50^. Therefore, these data suggest that this shift in methylation patterns mediated by integrin α6β4 is indeed a mechanism driving gene expression and progression to a more malignant phenotype in pancreatic cancer cells. While other evidence exists to suggest that the tumor microenvironment can influence epigenetics^47, 51, 52^, this study is the first to identify a specific mediator of the microenvironment, the integrin α6β4, as a regulator of this process.

Mounting evidence places the BER pathway as the most common, and context dependent mediator for active DNA demethylation^45, 53^. Our data support this concept, as we have demonstrated that modulation of multiple components of the BER pathway, including GADD45A, TET1, TDG, and PARP-1, impact transcriptional upregulation of AREG and EREG. Additionally, our confirmation that AREG and EREG enhancers become demethylated downstream of integrin α6β4, supports active DNA demethylation by DNA repair as the mechanism for transcriptional upregulation by the integrin α6β4. More specially, GADD45A acts as an important step in the activation of AREG and EREG and in accordance with the literature, is the coordinating molecule for specific DNA demethylation by BER^54^. We also show that recruitment of TDG to the nucleus is amplified in cells with high integrin α6β4 expression, suggesting that the integrin coordinates steps in this pathway, potentially through nuclear recruitment or specific targeting of repair factors. These data implicate the integrin α6β4 as a critical amplifier of DNA repair mediated DNA demethylation, identifying a novel mode of transcriptional upregulation in response to this integrin. Finally, we find that not only can the integrin α6β4 utilize BER to promote transcriptional upregulation also enhances the ability of pancreatic cancer cells to respond to and survive in the presence of DNA damage mediated by damaging agents whose damage is repaired by both the BER and NER pathways. This observation supports previous studies demonstrating that tissue architecture mediated by integrin α6β4 promotes resolution of double strand breaks^55^. Taken together these studies demonstrate that the integrin α6β4 contributes to a multitude of DNA repair pathways, and is a key component for connecting the extracellular environment with enhanced DNA repair.

In conclusion, this study examines a specific sensor of the tumor microenvironment, the integrin α6β4, and provides an exciting new role for this molecule in promoting tumor progression. Our data offer a novel mechanism for the upregulation of tumor promoting genes, alterations in the epigenome, and utilization of DNA repair, and places the integrin α6β4 as a major player in cancer epigenetics. These findings have far reaching impacts on our understanding of pancreatic carcinoma and further analysis of the integrin α6β4’s role in these processes will yield a more comprehensive understanding for how this integrin impacts tumor progression.

## Methods

### Cell lines and cell culturing

Panc1 cells (ATCC) were grown in Dulbecco’s modified Eagle’s medium (high glucose). Panc1 clones 2G6 (low integrin α6β4) and 3D7 (high integrin α6β4) were characterized and cultured as described previously^56^. Suit2 (Dr. Takeshi Iwamura, Miyazaki Medical College, Japan) and AsPC1 cells (America Type Culture Collection, ATCC) were maintained in RPMI 1640. Media was supplemented with 10% Fetal Bovine Serum (Sigma-Aldrich, St. Louis, MO), 1% penicillin, 1% streptomycin, and 1% L-glutamine (GIBCO by Life Technologies, Grand Island, NY).

### Immunocytochemistry (ICC)

Glass coverslips were coated with 10 μg/ml collagen I (BD Biosciences) at 4 °C overnight, then rinsed three times with PBS. Cells were plated on coverslips in normal culture medium and allowed to adhere for 4 hours before fixation. Cells were fixed, permeabilized, and immunostained as described previously^57^. Briefly, cells were fixed for 15 minutes with 4% paraformaldehyde containing 10 mM PIPES, pH 6.8, 2 mM EGTA, 2 mM MgCl_2_, 7% sucrose and 100 mM KCl for 15 min at room temperature, and permeabilized with 0.25% Triton X-100. Cells were blocked for 1 hour with 3% BSA + 1% goat serum in PBS. The following primary antibodies were used at indicated concentrations and incubated at 4 °C overnight: rat anti-CD104 (439-9B, BD Pharmingen, 1:100) mouse anti-Laminin-5 (γ2 chain, clone D4B5, Millipore, 1:500 dilution) in 3% BSA in PBST overnight at 4 °C. Cy3-conjugated goat anti-rat and Cy2-conjugated goat anti-mouse (Jackson Immuno Research, 1:500) and Alexa Fluor 647 phalloidin were incubated with 3% + 1% goat serum BSA for 1 hour at room temperature in dark. Coverslips were mounted on glass slides using 50% glycerol solution and sealed with clear polish. Images were acquired by total internal reflection (TIRF) microscopy using a Nikon Eclipse Ti. Images were processed for colocalization analysis and Pearson’s correlation coefficient by NIS Elements AR 3.2 software.

### Drug Treatment

5-Aza-2′deoxycytidine (5-aza-CdR; Sigma-Aldrich) and S-adenosylmethionine (SAM; NEB, Ipswich, MA) was added to cells in fresh medium daily at indicated concentrations for 3 or 5 days or equal volume DMSO or 0.005 M H_2_SO_4_ plus 10% ETOH respectively. JQ1 (250–500 nM; Bradner Lab; Dana-Farber Cancer Institute) or DMSO was added to cells for 16 hours. Gemcitabine (Sigma-Aldrich) or 3,4-Dihydro-5-[4-(1-piperidinyl)butoxyl]-1(2 H)-isoquinolinone (DPQ; Sigma-Aldrich) treatment or equal volume vehicle control was added once for 72 hours.

For H_2_O_2_ treatment (Sigma-Aldrich), cells were plated in a 96-well plate at 2000 cells/well. Medium was changed each day to normal growth 500 μM H_2_O_2_ containing medium. Cell density was measured using 3-(4,5-Dimethylthiazol-2-yl)-2,5-diphenyl-2H-tetrazolium bromide (MTT; Fisher Scientific) according to manufacturer’s protocol.

### RNA Extraction and Real-Time Quantitative PCR (QPCR)

Total RNA was extracted using Trizol reagent (Invitrogen, Carlsbad, CA) per manufacturer’s protocol. cDNA was synthesized using the High Capacity cDNA Reverse Transcription Kit (Applied Biosystems, Foster City, CA) and target expression was assessed using available probes, reagents, and the StepOnePlus Real-Time PCR System from Applied Biosystems, as performed previously^13^. Target expression (2^−ΔΔCT^) was normalized to endogenous reference (18S or β-actin) and reported relative to control samples. Each QPCR experiment was performed a minimum of three times and consistent trends across biologically replicated experiments were observed. The representative experiments shown in most figures are from experiments that generally best reflected the average QPCR data of these three experiments. We chose representative data over averaging the individual experiments together since significant variability often existed between experiments. This variability occurred because of the amplification of small differences present in the mRNA when values of one of the conditions are exceptionally low (as we see in the Panc-2G6 cells with AREG and EREG expression). The exceptions are data from Figs 4B,H and 5F, which showed less inter-experimental variability and were averaged in order to demonstrate significance of the findings.

### Whole Genome Bisulfite Sequencing

Whole genomic DNA was isolated from cell lines using the GenElute Mammalian Genomic DNA Miniprep Kit (Sigma-Aldrich). DNA was processed for high-resolution methyl-seq by the NextGen Sequencing Core at the Norris Comprehensive Cancer Center. Whole genome sequencing was done on an Illumina NextSeq and each library sequenced with paired-end runs for 150-bp read length analysis.

### Read Alignment and Differential Methylation Analysis

DNA reads were aligned against GRCH37 using Bismark^58^ software version 0.14.3, permitting at most one mismatch, considering both sequence and bisulfite conversion mismatches. Methylation calls for each CpG were extracted using Bismark methylation extractor tool. Read alignment revealed that many reads could be mapped to both AREG and the AREG pseudogene due to high degree of homology. To allow mapping of AREG, the AREG pseudogene was masked during analysis, and vice-versa. Differential methylation analysis comparing Panc1-3D7 and Panc1-2G6 was performed using Bioconductor DSS software version 2.10.0^59^. Differentially methylated loci (DML) were determined by >0.99 posterior probability of the difference in mean methylation levels being >0.3. Differentially methylated regions (DMR) were also detected by joining DMLs with p-value less than 0.01. DMRs have a minimum length >50 bps, minimum number of DML >3 and >50% of CpG sites with p-value < 0.01. DMRs with distance less than 100 bps were merged. DMLs and DMRs were annotated using methylKit^60^ version 0.9.5, where we defined the promoters as +/− 1000 bp from TSS and CpG shores +/− 2000 bp flanking each side of the CpG island.

### Gene knockdown by RNAi

For siRNA treatment cells (3 × 10^6^) were electroporated without or with 200 nM non-targeting or specific siRNA (Dharmacon, Inc.) as described previously^12^.

For shRNA, lentivirus was produced by combining MISSION constructs for packaging (psPAX2), envelope (pDM2G) and targeting shRNA or a non-targeting vector (pLKO.1), at a 4:2:1 ratio (Sigma Aldrich, St. Louis, MO). Polyethylenimine (PEI; Polysciences) was combined with DNA at a 3:1 ratio, and added to 70% confluent HEK 293LTV cells. Conditioned media was collected 24 and 48 hrs post transfection by centrifugation, and viral supernatant added to cells with 8 μg/ml hexadimethrine bromide (polybrene, Sigma-Aldrich). Gene expression was measured by QPCR 24 hrs following puromycin selection (2 μg/ml).

### Western Blotting Analysis

Cells were washed 2x with cold PBS and collected with 400 μl cold Buffer A (10 mM Hepes pH 7.9, 10 mM KCl, 0.1 mM EDTA, 0.1 mM EGTA, 1 mM DTT, 0.5 mM PMSF). Nuclei were lysed using 10% NP-40 and nuclear pellet resuspended in cold Buffer C (20 mM Hepes pH 7.9, 0.4 M NaCl, 1 mM EDTA, 1 mM EGTA, 1 mM DTT, 1 mM PMSF). Nuclear extracts were collected by centrifugation, separated using 10% SDS-PAGE, transferred and immunoblotted for TDG (Genetex, GT622), and Lamin A/C (EMD Millipore). Alternatively, whole cell lysates were collected using RIPA buffer, separated using 10% or 12.5% SDS-PAGE and then immunoblotted for integrin β4 (BD Transduction Labs, #611232), laminin γ2 chain for laminin-5 (Millipore, clone D4B5) or β-actin (Sigma-Aldrich). Uncropped﻿ western blot images can be found in the supplemental information.

### DNA Repair Analysis

Immuno-slot-blot analysis was performed as described previously^61^. Briefly, cells (70% confluent) were exposed to 30 J/m^2^ UV and harvested immediately or indicated times post-treatment. Cells were lysed with 10 mM Tris pH 8.0, 1 mM EDTA, 0.05% SDS, 100 μg/ml fresh proteinase K) and DNA isolated. DNA was bound to a nitrocellulose membrane using a slot blot apparatus and probed using antibody for 6-4 photoproducts (6-4 PP; Cosmobio). Data are reported as percent repair compared to the amount of initial damage (0 hr time point).

## Electronic supplementary material

Supplementary Figures
