# Supplementary material for: Integrin α6β4 Upregulates Amphiregulin and Epiregulin through Base Excision Repair-Mediated DNA Demethylation and Promotes Genome-wide DNA Hypomethylation
Source: Sci Rep. 2017 Jul 21;7:6174. doi: 10.1038/s41598-017-06351-4 (PMC5522472; doi:10.1038/s41598-017-06351-4)
Supplement: Supplementary file 1 — Supplementary Figures [file 41598_2017_6351_MOESM1_ESM.pdf]

Supplemental Information

Integrin α6β4 Upregulates Amphiregulin and Epiregulin through Base Excision Repair-Mediated DNA Demethylation and Promotes Genome-wide DNA Hypomethylation

Brittany L. Carpenter [1, 2], Jinpeng Liu[1], Lei Qi[1,2], Chi Wang[1, 3], and Kathleen L. O'Connor [1, 2, *]

[1] Markey Cancer Center, [2] Department of Molecular and Cellular Biochemistry, [3]Division of Cancer Biostatistics, Department of Biostatistics, University of Kentucky, Lexington, USA 40506-0509

[*]To whom correspondence should be addressed:
Kathleen L. O'Connor, Ph.D.
Markey Cancer Center
University of Kentucky
741 S. Limestone Street
Lexington, KY 40506-0509 USA
Phone: 859-323-7534 (office)
Fax: 859-323-6030
E-mail: kloconnor@uky.edu

A. Figure 1D. Integrin β4 immunoblot

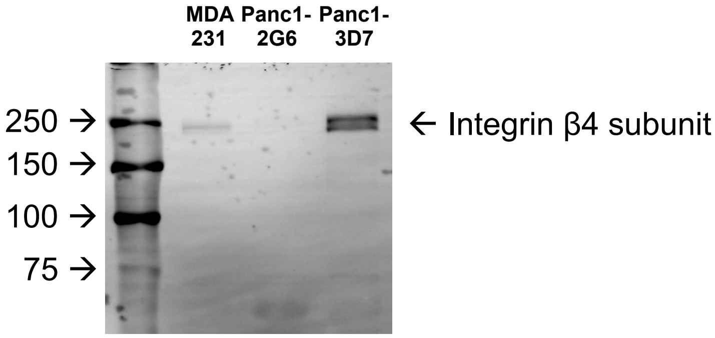

B. Figure 1D. Laminin-5 immunoblot

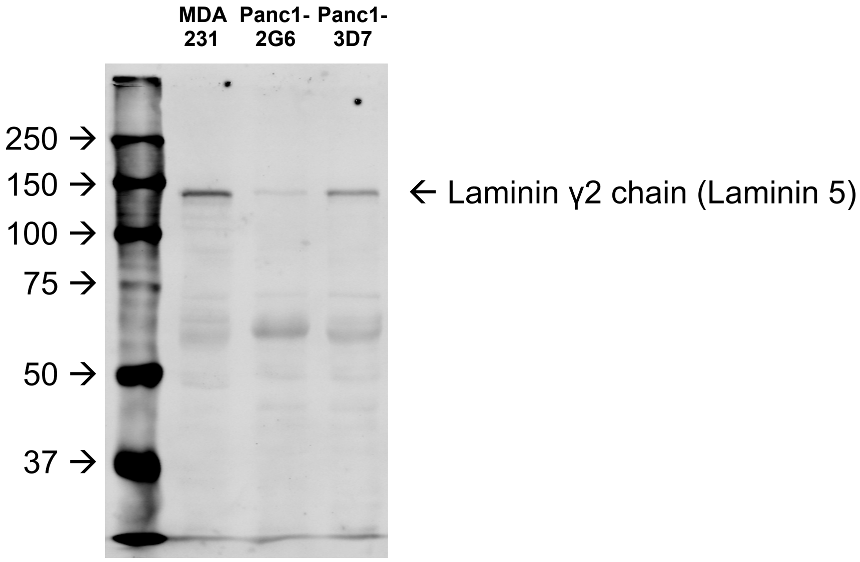

C. Figure 1D. Actin immunoblot

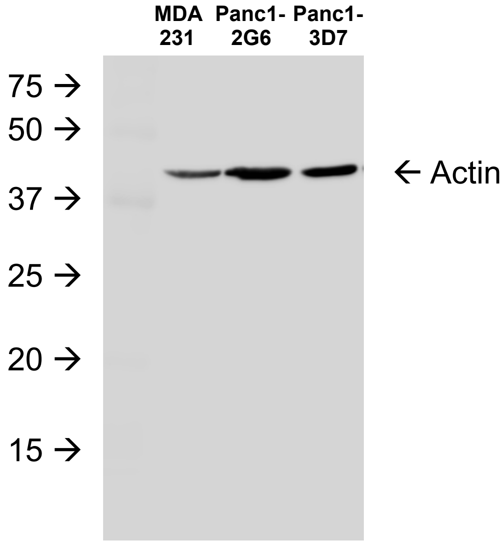

Figure S1. Full length original images for immunoblots in Figure 1D.

A. Figure 6C. TDG immunoblot

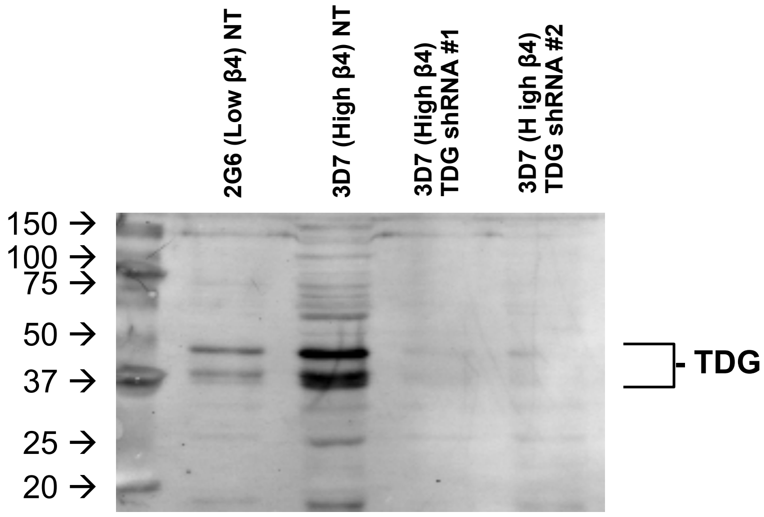

B. Figure 6C. Lamin A/C immunoblot

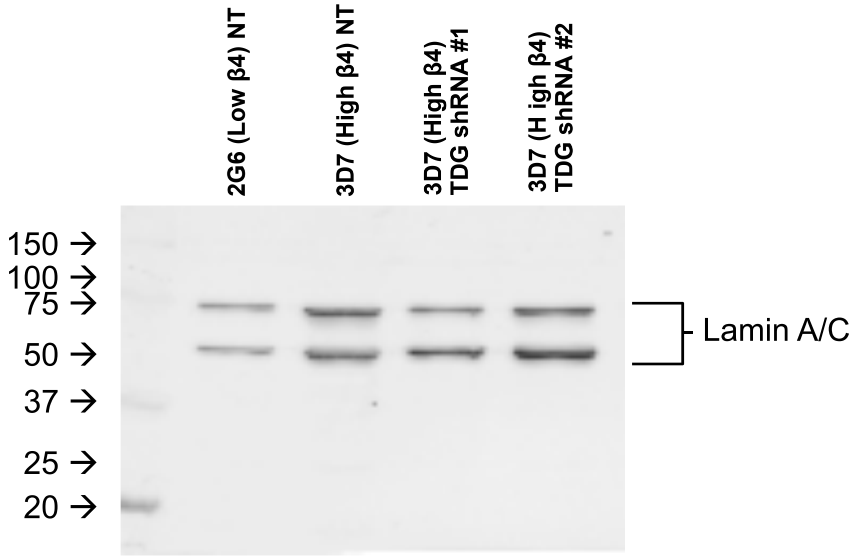

Figure S2. Full length original images for immunoblots in Figure 6C.
